# From the Allergic Cascade to the Epithelium-Driven Disease: The Long Road of Bronchial Asthma

**DOI:** 10.3390/ijms24032716

**Published:** 2023-02-01

**Authors:** Christian Domingo, Rosa M. Mirapeix

**Affiliations:** 1 Servei de Pneumologia, Hospital Parc Taulí de Sabadell, 08208 Sabadell, Spain; 2 Departament de Medicina, Universitat Autònoma de Barcelona (UAB), 08193 Barcelona, Spain; 3 Departament de Ciències Morfològiques, Universitat Autònoma de Barcelona (UAB), 08193 Barcelona, Spain

In medicine, much of the progress made is due to the emergence of new drugs. We learn the underlying mechanisms behind the modification or inhibition of certain pathways, as well as what occurs due to the lack of intervention. A clear example is the evolution in the knowledge of the pathophysiology of bronchial asthma.

In the second half of the last century, the boundaries of the traditional classification of allergic and non-allergic asthma were expanded [1]. Coinciding with the advent of immunotherapy, researchers revealed how the so-called allergy cascade was set in motion, that is, all the reactions that began with the activation of dendritic cells or antigen-presenting cells (APC). The presence of allergens, antigens that favour the production and release of immunoglobulin E (IgE) into the environment by plasma cells, promotes the activation of the Th2 pathway. The Th2 pathway requires the differentiation of Th0 lymphocytes (naïve T cells) into Th2 lymphocytes. This pathway can generate IgE against each allergen to which these antibodies become sensitised, hence called adaptive immunity (i.e., the production of the type of antibody “adapts” to the different allergens with which the individual has been in contact) [2]. Since the cell initiating the reaction is the Th2 lymphocyte, it is also called the Th2 pathway. Immunotherapy modifies the immune response and diverts it to the Th1 pathway instead of the Th2 pathway (as occurs in allergies) through its effect on regulatory T cells.

Subsequently, the first of several drugs called biologics (thus named because they were originally derived from live Chinese hamster cells), or monoclonal antibodies (mAbs), was marketed. The first of these blocked IgE, thus acting again on the Th2 pathway. Allergen-sensitised, IgE-producing patients with clinical symptoms as a consequence of allergen exposure were referred to as patients with allergic phenotypes [3,4].

Posteriorly, it became known that during this Th2 process, other molecules known as interleukins (IL5, IL4, and IL13), were released into the environment [5]. It has been confirmed that the presence of these interleukins favours respectively the synthesis, maturation, and recruitment of eosinophils; the activation of the Th2 pathway and the blocking of VCAM-1 expression; and finally, the activation of INOs, the enzyme that favours the release of the exhaled fraction of nitric oxide (FENO). The presence of these molecules increases in certain pathological situations and are, therefore, markers of disease (biomarkers). Like IgE, these interleukins, as well as eosinophils, were initially observed in the Th2 pathway, but later, it was noted that after blocking IgE and downregulating the Th2 pathway, they continued to be present. At that time, it was documented that the activation of other cells, such as type 2 innate lymphoid cells (ILC2) and natural killer T cells (NKT cells), also led to an increase in the same ILs as well as eosinophils. It was, therefore, revealed that the production of these ILs might occur as a consequence of the activation of more than one pathway. ILC2 and NKT cells are cells of the so-called innate immune system and respond in the same way regardless of the noxa; thus, there is no “adaptive response” to the type of trigger. Patients with elevated eosinophil levels were defined as those with eosinophilic phenotypes. Since immunological factors are not dichotomous, there is a third group of patients who have biomarkers of both pathways. Patients with elevated biomarkers (allergic and/or eosinophilic phenotype) caused by the activation of Th2 lymphocytes and/or ILC2 were considered as those with T2 inflammation and by exclusion, all other patients with asthma who did not have a T2 phenotype were considered to have non-T2 asthma.

More recently, it has been observed that the activation of innate immunity is produced by other molecules, namely TSLP, IL33, and IL25 [5,6]. Without discussing the characteristics of each of these molecules in detail, it was observed that they appear as a result of damage to the bronchial ciliated epithelium, which, in these circumstances, releases these molecules into the environment. Since they represent an alarm signal indicating epithelial damage (epithelial cell disruption, the appearance of intercellular space, loss of cilia, the thickness of basement membrane, and in general, loss of the barrier function of the epithelium), they are called alarmins. These alarmins directly activate the cells of innate immunity. Blocking them with a new anti-TSLP antibody has shown that these alarmins, especially TSLP, exert control over the activity of antigen-presenting cells. Disease control is, therefore, exerted by the bronchial ciliated epithelium, which releases alarmins into the environment when it is damaged. T2 asthma (and perhaps non-T2 asthma) then becomes an epithelium-driven disease. As mentioned above, anti-alarmin mAbs are currently being developed. It should be kept in mind, however, that in immunology, and in the human body in general, there are always regulatory mechanisms that exert functions antagonistic to physiological ones in an attempt to achieve homeostasis. When we administer a mAb, we are modifying the phenotype of the individual, and this causes their body to try to maintain the lost balance by activating other pathways. An example is the activation of the Th2 pathway by allergens, through already-sensitised B cells that express IgE on their surface. Although this allergic pathway is stopped at the level of APCs, the possibility exists that it may be activated by the activation of a lower loop. Figure A1 illustrates these pathways.

In this brief account, we described the evolution of our understanding of bronchial asthma. Owing to the emergence of various drugs with immunoregulatory effects, we have moved from the now-distant concept of the “allergic cascade” to that of epithelial-driven disease. We shall see what the future holds.

## Figures and Tables

**Figure A1:**
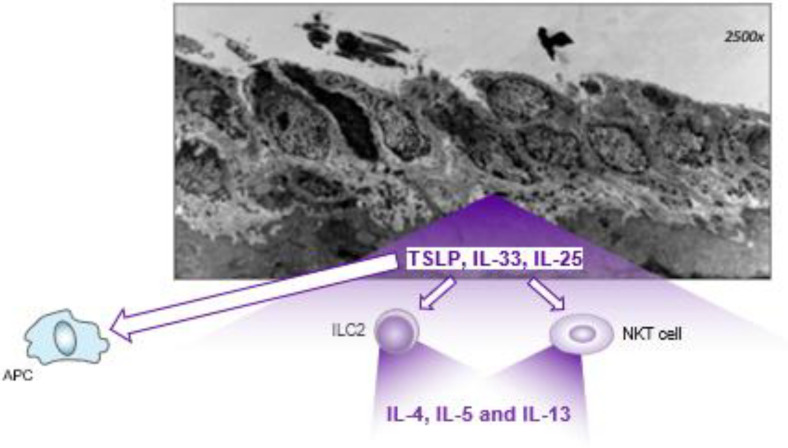
This figure shows the disruption of the ciliated bronchial epithelium that favours the release of alarmins. Alarmins, in turn, activate, below the epithelium, the innate immunity responsible for the initial production and release of the three interleukins (IL5, IL4, and IL13) which contribute to the definition of the T2 inflammation. On the left upper side, the arrow shows the regulatory effect of TSLP on adaptive immunity (partially modified from Domingo C.; *Drugs* 2017 [5]).
